# CD73 Expression Is Dynamically Regulated in the Germinal Center and Bone Marrow Plasma Cells Are Diminished in Its Absence

**DOI:** 10.1371/journal.pone.0092009

**Published:** 2014-03-24

**Authors:** Laura J. Conter, Eunice Song, Mark J. Shlomchik, Mary M. Tomayko

**Affiliations:** 1 Department of Dermatology, Yale University School of Medicine, New Haven, Connecticut, United States of America; 2 Department of Immunobiology, Yale University School of Medicine, New Haven, Connecticut, United States of America; 3 Department of Laboratory Medicine, Yale University School of Medicine, New Haven, Connecticut, United States of America; COCHIN INSTITUTE, Institut National de la Santé et de la Recherche Médicale, France

## Abstract

CD73 catalyzes the conversion of extracellular nucleosides to adenosine, modulating inflammatory and T cell responses. Elevated expression of CD73 marks subpopulations of murine memory B cells (MBC), but its role in memory development or function is unknown. Here, we demonstrate that CD73 is progressively upregulated on germinal center (GC) B cells following immunization, is expressed at even higher levels among T follicular helper cells, but is absent among plasma cells (PC) and plasmablasts (PB). We analyzed the T-dependent B cell response in CD73 knockout mice (CD73KO). During the early response, CD73KO and wild type (WT) mice formed GCs, MBCs and splenic PBs and PCs similarly, and MBCs functioned similarly in the early secondary response. Late in the primary response, however, bone marrow (BM) PCs were markedly decreased in CD73KO animals. Tracking this phenotype, we found that CD73 expression was required on BM-derived cells for optimal BM PC responses. However, deletion of CD73 from either B or T lymphocytes alone did not recapitulate the phenotype. This suggests that CD73 expression is sufficient on either cell type, consistent with its function as an ectoenzyme. Together, these findings suggest that CD73-dependent adenosine signaling is prominent in the mature GC and required for establishment of the long-lived PC compartment, thus identifying a novel role for CD73 in humoral immunity.

## Introduction

CD73, ecto-5′ - nucleotidase, is a glycosylphosphatidylinositol-linked surface glycoprotein that plays a rate-limiting role regulating extracellular ATP and adenosine levels [1]–[3]. ATP is released into the extracellular space after tissue injury or inflammation and functions as a danger signal. CD39 converts ATP to AMP, and CD73 dephosphorylates AMP to adenosine. Adenosine binds to and signals through specific GPCRs to alter intracellular cAMP levels and control inflammation and vascular permeability [4].

CD73 is widely expressed, including on hematopoietic cells and endothelial cells, and is induced in response to cellular stress, hypoxia and inflammation [2], [4]. Mice lacking CD73 have exaggerated deleterious responses to diverse stresses, including enhanced vascular leakage following hypoxia [5], pulmonary injury from bleomycin [6], joint swelling after Borrelia infection [7] and mortality following microbial sepsis [8].

In the immune system, the predominant adenosine receptor is A2a and its ligation modulates the function of activated T cells [9], dendritic cells (DCs), neutrophils and macrophages [2], [10]. CD73 and CD39 are expressed by CD4^+^ CD25^+^ FoxP3^+^ T regulatory cells in mice [11], [12] and humans [13]. Extracellular adenosine generated by these T regulatory cells binds A2a receptors on activated effector T cells suppressing proliferation [11], [14]. CD73 and CD39 are overexpressed in many cancer cells and function to suppress anti-tumor T cell responses via their adenosine production [15]–[17]. Conversely, loss of CD73 protects against tumor metastasis [18], [19].

CD73 caught our attention when we discovered it was upregulated on a subset of murine MBCs [20]. Prior to this, Thompson and colleagues had postulated that CD73 expression was associated with a memory state, observing that it was expressed at low levels among human neonatal B cells but upregulated in infant B cells preceding the onset of IgG responses and that it was upregulated among IgG-switched B cells in the tonsil [21]–[23]. Recently, CD73 upregulation among antigen-experienced [24] and GC B cells [25] has been described, supporting the notion that CD73 activity is associated with MBC formation. Overall, however, little is known about its function in humoral responses.

Here, we asked what consequence loss of CD73 function has on the development of the T–dependent B cell response in mice. We show that CD73 expression is tightly modulated during the B cell response, increasing within the GC on B and T_FH_ cells over time, and that intact CD73 activity is required for the establishment of a normally-sized BM PC compartment. For an optimally sized BM PC pool, we further found that expression on either B cells or T cells themselves is not required, although expression on hematopoietically derived cells is, consistent with the notion that CD73 works in trans, generating adenosine in the extracellular milieu. Together, the data suggest that CD73 activity in the late GC enhances the formation or maintenance of BM PCs.

## Materials and Methods

### Ethics statement

All studies were carried out in strict accordance with the recommendations in the Guide for the Care and Use of Laboratory Animals of the National Institutes of Health. The protocol was approved by the Yale Institutional Animal Care and Use Committee (protocol number 07628). All efforts were made to minimize suffering.

### Mice, immunizations, adoptive transfers and BM chimeras

CD73 targeted deletion mice (CD73KO) [26], a gift of Jurgen Schnermann, were backcrossed to C57Bl/6J (B6) mice for 10 generations then bred to homozygosity for the CD73 deletion. B6.129P2-*Tcrb^tm1Mom/J^* (TCRbetaKO), B6.129S2-*Ighm^tm1Cgn/J^* (muMT), and C57Bl/6J mice were purchased from the Jackson Laboratory (Bar Harbor, ME) then bred and maintained in our laboratory as described [27], [28]. B6Ly5.2Cr (CD45.1) B6 and C57Bl/6 (CD45.2) mice for non-breeding experiments were purchased from the National Cancer Institute.

For immunizations, mice were given i.p. injections of NP_32_-chicken gamma-globulin (4-hydroxy-3-nitrophenyl) acetyl chicken gamma-globulin (NP-CGG) precipitated in alum, as described [27].

For adoptive transfer in secondary response experiments, splenocytes were depleted of T cells by magnetic-bead-mediated negative selection (Stemcell Technologies, Vancouver, British Columbia, Canada). Flow cytometry was used to confirm that T cell depletion was >90% and to standardize precursor numbers for transfer. Recipients were B6Ly5.2Cr (CD45.1). Endogenous responding B cells in these recipients can be distinguished from donor B cells based on surface expression of the CD45 allele. Recipients were immunized 16-hours post transfer, as described above.

For mixed BM chimera experiments, CD45.1.B6 or CD73KO recipient mice were treated with 2 individual 450 RAD doses of cesium irradiation spaced three hours apart. 1-hour later, donor bone marrow was transferred i.v. via tail vein. Six weeks post transfer, recipients were immunized with NP-CGG in alum i.p, as described above. Drinking water was supplemented continuously from transfer to sacrifice with Baytril 100 0.33 mg/ml (enrofloxacin, Bayer Healthcare LLC, Animal Health Division, Shawnee Mission, KA).

### Purification and analysis of cells by flow cytometry

Single cell suspensions of RBC-depleted splenocytes were stained for flow cytometric analysis [27], [29], analyzed on an LSRII and/or sorted on a FACSAria (BD Immunocytometry Systems). Data were analyzed with FlowJo (Tree Star), as described [27]. Gating strategies for B cell subpopulations and T_FH_ cells are demonstrated in Figure S1. Total numbers of events collected ranged from 1–10 million per sample, with more events collected for analysis of rare populations.

Anti-kappa (187.1)-Pacific Blue, -CD19 (1D3.2)-Pacific Blue, -CD4 (GK1.5)-Pacific Blue, -CD317/BST-2 (eBio927)-biotin, -PD1/CD279 (G4)-biotin, -CD80 (16-10A1)-biotin, -Ly6G (1A8)-Fitc, -Alexa-647 and -biotin, -F4/80 (F4/80)-Alexa-647 and -Alexa-488, -CD11c (M1/70)-Alexa-647, -CD44 (1M7)-Alexa-647, -CD62L (Mel-14)-Alexa-680, labeled-(4-hydroxy-5-iodo-3-nitrophenyl)acetyl (NIP) reagents and streptavidin conjugated Alexa-647 and Alexa-488 were produced in our laboratory. Anti-IgG_1_ (A85-1)-Fitc, -CD73 (TY/23)-PE, -CD38 (90)-PE and -biotin, -CD45R/B220 (RA3-6B2)-APC/Cy7, -CD19 (1D3)-APC/Cy7, -CD95/Fas (Jo2)-PE/Cy7, -CD138/syndecan (281-2)-biotin, -CD185 (2G8)-PE, -CD25 (PC61)-PE/Cy7, -CD162/PSGL1 (2PH1)-PE, -Siglec-F (E50-240)-Alexa-647 and streptavidin conjugated PE/Cy7 and APC/Cy7 were from BD Biosciences (San Jose, CA). Anti- -CD45.2 (104)-Pacific Blue, -PDL2/CD273 (TY-23)-biotin, -CD80 (16-10A1)-PE, -CD3epsilon (145-2C11)-Fitc, -Siglec H (eBio440c)-Alexa-647, FoxP3 (FJK-16s)-APC and –Cxcr4/CD184 (2B11)-PE were from eBioscience, Inc. (San Diego, CA). Anti-CD279/PD-1 (29F.1A12)-PE, CD279/PD-1 (RMP1-30)-PE/Cy7 and -biotin, -CD278/ICOS (C398.4A)-Fitc, -CD44 (1M7)-APC/Cy7, -CCR7 (4B12)-biotin, -TCRb (H57-597)-PE/Cy7 and APC/Cy7, -CD11c (N418)-PE/Cy7 and -Brilliant Violet, -CD11b (M1/70)-APC/Cy7 and –Brilliant Violet, -I-A/I-E (M5/114.15.2)-Fitc and –PE,–CD38 (90)-PE and –CD49b (DX5)-biotin, CD18-PE and -IgE (RME-1)-Fitc were from Biolegend, Inc. (San Diego, CA). F(ab′)2 fragment goat anti-human IgG conjugated to PE was from Jackson ImmunoResearh (West Grove, PA). Ethidium monoazide (EMA) and propidium iodide (PI) were from Invitrogen (Carlsbad, CA).

### Proliferation assays

Mice were given i.p injections of 3 mg BrdU (Sigma Aldrich) i.p. 1-hour prior to sacrifice. As described [30], [31], cells were stained for surface markers, permeabilized with ethanol, fixed with paraformaldehyde and Tween 20 then treated with 100 Kunitz units of DNAse (Sigma-Aldrich). Then cells were stained with anti-BrdU (PRB-1)-Fitc (Phoenix Flow Systems, San Diego, CA) and analyzed by flow cytometry, as described above.

### Apoptosis assays

Cells were incubated with a Fitc-conjugated pan-caspase inhibitor, VAD(OMe)-FMK (SM Biochemicals LLC, Anaheim, CA) in RPMI 1640 for 45 min at 37°C and washed as described [31]. Cells were then stained with labeled antibodies to surface proteins and analyzed by flow cytometry, as described above.

### ELISpot assays

Immulon 4HBX plates (Fisher Scientific, Pittsburgh, PA) were coated with 5 µg NP_16_- or NP_2_-BSA, and nonspecific binding blocked with 1% BSA in PBS. Lymphocytes were plated in triplicate at serial dilutions then incubated for 5 hours at 37°C. Plates were washed and stained with anti-IgG_1_ (1070-04)-alkaline phosphatase secondary antibody (Southern Biotech) and developed with 5-Bromo-4-chloro-3-indolyl phosphate (BCIP, Southern Biotech). Spots were counted manually at the dilution that yielded 25–50 spots per well.

### V region sequencing

DNA was prepared from FACS purified cells and V-lambda_1_ sequences amplified by nested PCR as described [32]. PCR products were cloned into PCR-TOPO-blunt vector (Invitrogen) and inserts in individual colonies were sequenced and aligned to a rearranged germline V-lambda_1_/J-lambda_1_sequence, as described [32]. Statistical analysis of replacement and silent mutations in the CDR and framework regions of the V-lambda_1_ sequences to detect selection was performed as described [33].

### Statistical analysis

Statistical significance was determined by the unpaired and paired Student's *t* test.

## Results

### CD73 is expressed in the GC and increases with maturation

**Figure 1:**
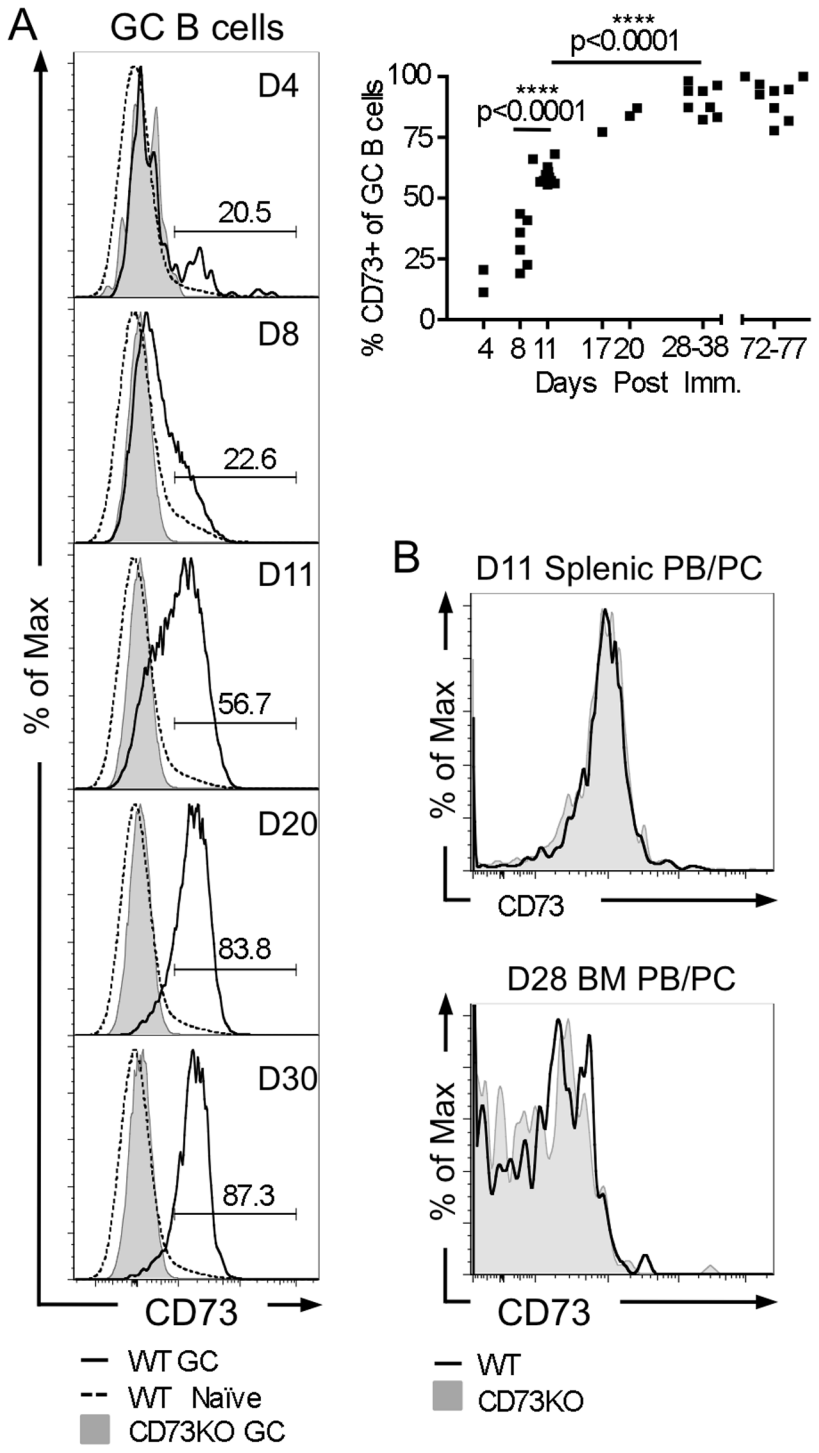
CD73 expression is modulated among responding antigen-specific B lineage cells in response to immunization with T-dependent antigen. Splenocytes and BM cells from B6 WT and control CD73KO mice were stained and analyzed by flow cytometry on the indicated days post i.p. immunization with NP-CGG in alum. Representative FACS histograms of CD73 expression are shown. 3 million events were collected per sample. (**A**) CD73 expression on splenic GC B cells from WT (solid line) or CD73 KO (shaded gray), identified as NIP^+^ kappa^lo^ CD38^low^ CD95^hi^ CD19^+^. For comparison, staining of WT kappa^+^ CD38^hi^ CD95^lo^ CD19^+^ cells, which are predominately naïve, is shown (dashed line). (**Upper right**) Percentage of CD73^+^ GC B cells as a function of time; each point represents an individual mouse. On days 72–77, GC B cells were gated as NIP^+^ kappa^lo^ CD95^hi^ CD19^+^ (without CD38 gating). (**B**) CD73 staining of PB/PC populations from the spleen and marrow of WT (solid line) and CD73KO (shaded gray) mice. Antigen specific splenic and BM PB/PC populations were identified as intracellular NIP_5_BSA^hi^ surface B220^−^, and were a mixture of CD138^hi^ and CD138^lo^ cells. Similar results were seen when gated on IgG_1_^hi^.

To characterize regulation of CD73 expression during the immune response, we evaluated CD73 expression by flow cytometry among B lineage cells in B6 WT and CD73KO mice immunized i.p. with NP-CGG. While naïve B cells were CD73-negative, GC B cells at early time points were comprised of CD73-negative and positive populations. With time, the fraction of GC B cells expressing CD73 increased, from an average of 31.1% at day 8 to 59.9% at day 11 (p<0.0001), to 90.4% by day 28–38 (p<0.0001) (Figure 1A.), confirming and extending recent observations by Kaji et al [25]. In contrast to GC B cells, PB and PC populations from both the spleen and marrow were uniformly negative for CD73 expression (Figure 1B). Thus, naïve B cells and splenic PBs, which arise primarily outside of the GC, are CD73-negative, while GC B cells and some of their MBC progeny are CD73-positive, but their BM-resident PCs progeny are CD73-negative. These findings may relate to observations that, when GC formation is inhibited, CD73-positive MBC but not CD73-negative MBC are diminished [24]. Thus, CD73 expression appears to be tightly regulated on B lineage cells during the response to T-dependent antigen.

**Figure 2:**
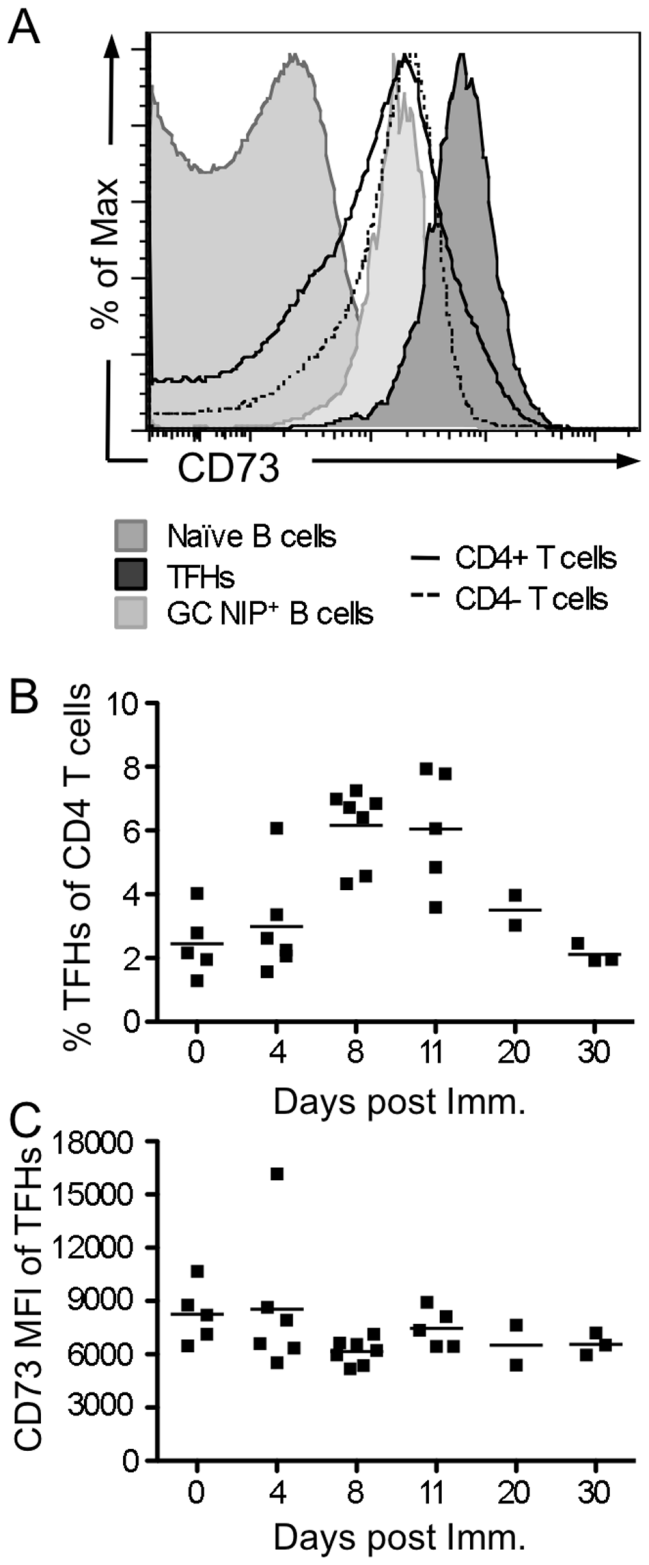
T_FH_ kinetics and CD73 expression. At the indicated days post i.p. immunization with NP-CGG in alum, spleens from B6 WT and CD73KO control mice were stained and analyzed by flow cytometry. T_FH_ were identified as TCRbeta^+^ CD4^+^ CD44^hi^ ICOS^hi^ PD-1^hi^. Similar results were observed with additional PSGL1^low^ gating. 3 million events were collected per sample. (**A**) Representative histogram showing high expression of CD73 on T_FH_ cells 11 days post immunization (shaded dark gray). For comparison, low expression on naive splenic CD19^+^ CD38^+^ CD95^−^ B cells (shaded medium gray), GC B cells (CD95^+^ CD38^−^ NIP^+^ CD19^+^ day 20 post-immunization, shaded light gray), total CD4^+^ T cells (solid black) and CD4^−^ (CD8^+^) T cells (dashed gray) is shown. (**B**) Kinetics of T_FH_ cell expansion post immunization. (**C**) Relative intensity of CD73 staining among T_FH_ cells at different times post immunization. Each point represents an individual mouse and bars are means.

As CD73 is expressed by CD8^+^ and some CD4^+^ T cells, including subsets of regulatory T cells that require its activity for immunomodulation [11]–[14], [34], [35], we asked if T cells participating in the GC response also express CD73. We found that T_FH_ cells, critical for GC formation and maintenance and for differentiation of PC and MBC [36], uniformly expressed very high amounts of CD73 (Figure 2A). The expression was higher than that observed among any other T or B lymphocyte subsets analyzed, including CD73^+^ GC B cells, MBCs, CD8^+^ T cells or non-T_FH_ CD4^+^ T cells (Figure 2). Expression on T_FH_ was not modulated in the weeks following immunization, although the frequency of T_FH_ cells increased (Figure 2B,C). Together, these data indicate that CD73 protein expression increases within the GC milieu during maturation, via both B and T cell sources, and suggest that the GC microenvironment is potentially rich in extracellular adenosine.

### CD73 is required for normal numbers of BM PCs in the late phase of the primary response

As long-lived PCs arise from precursors in the GC, particularly the late GC [37] when our data indicate CD73 expression is abundant, we postulated that CD73 might shape their development. Conversely, as short-lived splenic PBs that characterize the early primary response are derived largely outside of the GC, prior to increased CD73 expression, we postulated that CD73 was not important for their development. To test these interrelated hypotheses, we immunized CD73KO and WT mice with NP-CGG in alum and compared the kinetics and quality of the resultant splenic and BM PB and PCs by ELISpot.

**Figure 3:**
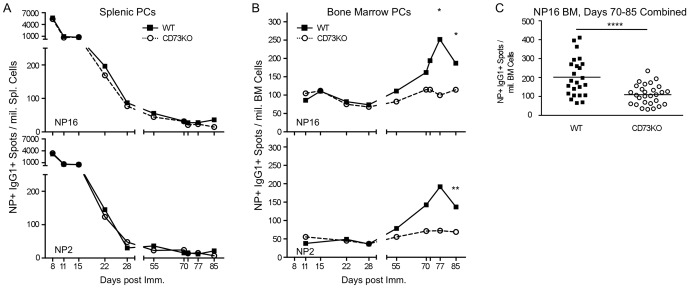
BM-resident PCs are diminished late in the primary response in the absence of CD73. On the indicated days post i.p. immunization with NP-CGG in alum, CD73KO (empty circles, dashed lines) or WT (filled squares, solid lines) spleen and BM were harvested for ELISpot analysis of NP-binding IgG_1_^+^ PB/PCs. (**A**) Splenic Elispots. (**B**) BM Elispots. In A and B, upper and lower panels show frequencies of NP16- and NP2-binding Elispots, respectively. Day 28 and day 11 represent 4 and 2 independent experiments, respectively, each with 4–10 individual mice. Each other point is averaged from 4–10 individual mice in a single experiment. Error bars depict standard deviations. The symbols *, ** and **** represent t test values of <0.05, <0.01 and <0.0001, respectively. BM NP_16_ paired t test for day 55–85 was 0.03. A day 72 BM NP_2_ value was excluded due to technical variation. (**C**) Complied BM NP_16_ day 70, 72, 77 and 85 ELISpots. Student's *t* test gave p<0.0001.

As predicted, there were no apparent differences in the kinetics or magnitude of the splenic PB response in the presence or absence of CD73 (Figure 3A). Relative affinities of the splenic PBs were also indistinguishable. In the early and peak response, the kinetics of PC accumulation in the BM were similarly unremarkable. However, in the late response, when the GC response has waned in size but is actively producing PCs [38], BM PCs in CD73KO animals were reduced (Figure 3B), plateauing at 0.54-fold of the WT response at days 70–85 (pooled day 70, 72, 77, 85 p<0.00011; Figure 3C). PC affinity, calculated by the ratio of NP_2_/NP_16_ ELISpot frequency, was unaltered. Similarly, BM PC surface expression of CXCR4, the beta-integrin CD18 and CD138, required for BM homing and retention [39]–[41], was unaltered (data not shown). Thus, in the first 3- to 4-weeks following immunization, formation and maintenance of PB and PC compartments in the spleen and BM appear to be independent of CD73 function, while after this time, CD73 is required for optimal BM PC generation, homing or maintenance. Since BM PCs themselves do not express CD73, these data suggest a requirement for CD73 activity among their GC precursors or among adjacent cells in the BM microenvironment.

To further determine potential sources of CD73 activity for incipient or formed BM PCs, we characterized CD73 expression among resident populations in the BM and spleen by flow cytometry. Eosinophils and basophils in the marrow, required for PC maintenance [42], [43], were uniformly CD73 negative (Figure S2). In the spleen, neutrophils expressed low amounts of CD73, while conventional and plasmacytoid dendritic cells (cDCs and pDCs) and macrophages [37] did not express appreciable surface CD73 (Figure S3A). CD73 levels were unaltered by immunization (data not shown). These findings are in keeping with previous observations by others that CD73 is not expressed by splenic myeloid cells [22] and that CD73 mRNA is inducible in neutrophils with LPS stimulation [44].

### Loss of BM PCs is dependent on CD73 expression by BM-derived cells

**Figure 4:**
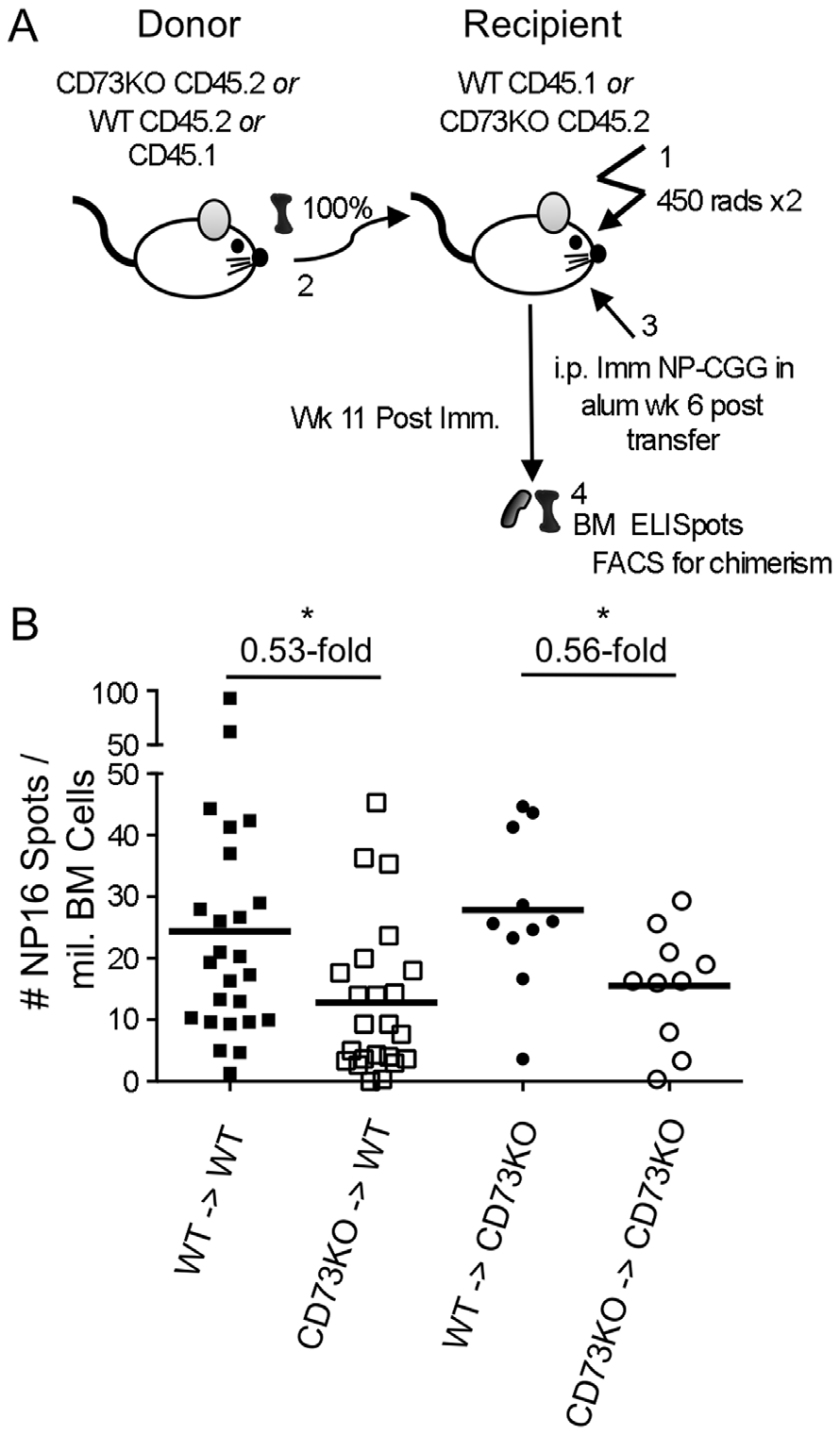
The BM PC compartment is diminished in the absence of BM-derived CD73. (**A**) Schematic of experimental design. Chimeric animals were established from adoptive transfer of WT or CD73KO donor BM into irradiated WT or CD73KO hosts. Donor and hosts were allotypically distinct (CD45.1 and CD45.2) in all chimeric combinations except the CD73KO donor/CD73KO host controls. 6-weeks post BM transfer, chimeric animals were immunized i.p. with NP-CGG in alum, and 11-weeks later, BM PCs were enumerated by ELISpot. Extent of chimerism was evaluated by flow cytometry analysis of splenocytes and is detailed in Figure S4 and in the text; 1–3 million events were collected per sample. (**B**) Evaluation of chimeric mice 11-weeks post-immunization. Frequency of IgG_1_ NP-specific PCs per million BM cells, determined by ELISpot analysis. Each point represents an individual mouse. Data shown are pooled from 6 (WT into WT), 5 (KO into WT) and 1 (WT into KO and KO into KO) individual experiments. Mean values are depicted by heavy horizontal lines. * indicates Student's t-test p values of <0.05.

CD73 is expressed on stromal cells, such as endothelia [5], [45], as well as hematopoietically-derived cells. To determine if the CD73 activity required for a normally sized BM PC compartment was hematopoietically-derived, we made reciprocal BM chimeras from CD73KO and WT strain mice (Figure 4A). To access chimera quality, donor and recipient B cells were distinguished by CD45 isotype, except for CD73KO-donor into CD73KO-recipient controls, where this was not possible. Chimeric mice were immunized with NP-CGG in alum i.p. and their BM PCs measured 11-weeks later. Donor B and T lymphocyte chimerism was effective and similar across groups (Figure S4). Donor-derived B cells accounted for an average of 96.0% (STD 3.5%), 94.6% (STD 5.6%) and 98.9% (STD 0.9%) in WT into WT controls, CD73KO into WT and WT into CD73KO mice, respectively, and donor-derived T cells accounted for an average of 79.5% (STD 4.7%), 79.3% (STD 6.1%) and 85.9% (STD 3.4%) in each of these groups.

Though BM PCs were detectable in chimeric mice 11-weeks post immunization at lower frequencies than in WT and CD73KO mice, presumably due to irradiation effects, we could compare between the groups that were treated equivalently. Indeed, the frequency of NP-specific IgG_1_ BM PCs was dependent upon donor BM CD73 genotype. NP_16_-binding PCs were less abundant among irradiated WT recipients of CD73KO compared with WT BM (0.53-fold, p = 0.0236) and less frequent among irradiated CD73KO recipients of CD73KO compared with WT BM (0.56-fold, p = 0.0239) (Figure 4B). Conversely, the host strain appeared to have minimal effect on PC frequency, as NP_16_-binding PCs were present at similar frequencies among irradiated CD73KO and WT recipients of WT BM and among irradiated CD73KO and WT recipients of CD73KO BM. Thus, CD73 expression among radiosensitive, hematopoietically-derived cells plays a dominant role in determining the size of the BM PC pool.

### The size of the BM PC compartment is not reduced by the isolated loss of either B or T cell-derived CD73

To determine if B cell-intrinsic CD73 activity was required for a normally sized BM PC pool, we made mixed BM chimeric mice in which the B cell compartment was derived from either CD73KO or CD73WT B6 donors and the remainder of hematopoietic lineages predominately from B cell deficient CD73WT muMT- donors (see Materials and Methods and Figure S5A). Chimeric mice were immunized with NP-CGG in alum i.p. and their BM PCs measured 11-weeks later. Donor B cell chimerism, assessed by CD45 isotype, was effective and similar in WT and CD73KO chimeras (average 96.4% (STD 2.3%) and 94.2% (STD 5.6%), respectively, Figure S5B). The frequency of NP-specific IgG_1_ BM PCs was indistinguishable between the two experimental groups (p = 0.5197) (Figure S5B). Together, these data indicate that when the rest of the BM compartment is derived from CD73-intact precursors, B cell-specific CD73 expression is not required to generate or maintain a normally sized BM PC pool.

Since CD73 expression on B cells was not required to mediate the reduction PC accumulation observed in CD73KO animals, and since T_FH_ cells are a robust source of CD73 within the GC, we asked if the global KO phenotype was dependent on T cell-specific CD73 expression. Mixed BM chimeric mice were established as described above, except that TCRbetaKO donor BM was mixed with WT or CD73KO BM (Figure S5A). After chimerism was established, mice were immunized and the BM PC compartment analyzed 11-weeks later. Again there were no differences in the frequencies of BM PCs (Figure S5B). Notably, chimerism was extensive but just under 80% (Figure S5B), significantly lower than seen in the B cell chimeric mice described above.

Together, these data indicate that CD73 expression specifically on B cells or T cells is not required for a normally sized BM PC pool. Rather, these data suggest a model wherein GC B cells, T_FH_ cells and possibly other BM-derived cell sources together contribute CD73 enzymatic activity in the intracellular space within the GC. This in turn is required to support the development of a robust BM PC compartment and ultimately optimal long-lived humoral immunity.

### The primary response is largely unremarkable in the absence of CD73

To further understand the role of CD73 activity in shaping the outcome of the B cell response, we examined the quality and kinetics of the primary response in the absence of CD73 in detail. Overall, the differences between CD73KO and WT mice were at most modest, suggesting that for many key events, CD73 activity is either dispensable or redundant. We will outline our findings briefly, chronologically from time of immunization.

### CD73 is required for splenic expansion after immunization with alum

At d11 post-immunization with NP-CGG in alum, CD73KO spleens were 1.25-fold (p = 0.014) reduced in weight compared with WT controls, though they were similar prior to immunization (Figure S6A). This reduced weight was presumably mainly due to fewer splenic RBCs in CD73KO mice, as total nucleated cells were similar in both groups (Figure S6B). Commensurately, there were no statistically significant differences in size of the splenic CD19^+^ B, CD3epsilon^+^ T or CD4^+^ T cell compartments in WT and CD73KO mice at baseline or post immunization (Figure S6C). The reduction in splenic RBCs in CD73 deficient mice may reflect a role for CD73-driven adenosine production in modulating vascular permeability during the immune response [5], [46].

We also evaluated conventional and plasmacytoid dendritic cell (cDC and pDC), macrophage and neutrophil compartments in the spleen. In general, the numbers of these cell types were unaffected in the absence of CD73, although at day 28 post immunization, there was a modest increase in cDC and decrease in neutrophils in CD73KO mice (Figure S3B), so changes in these population cannot account for decrease in weight observed in VD73-deficient spleens.

### Modest alterations in GC size, resolution kinetics and quality in the absence of CD73

**Figure 5:**
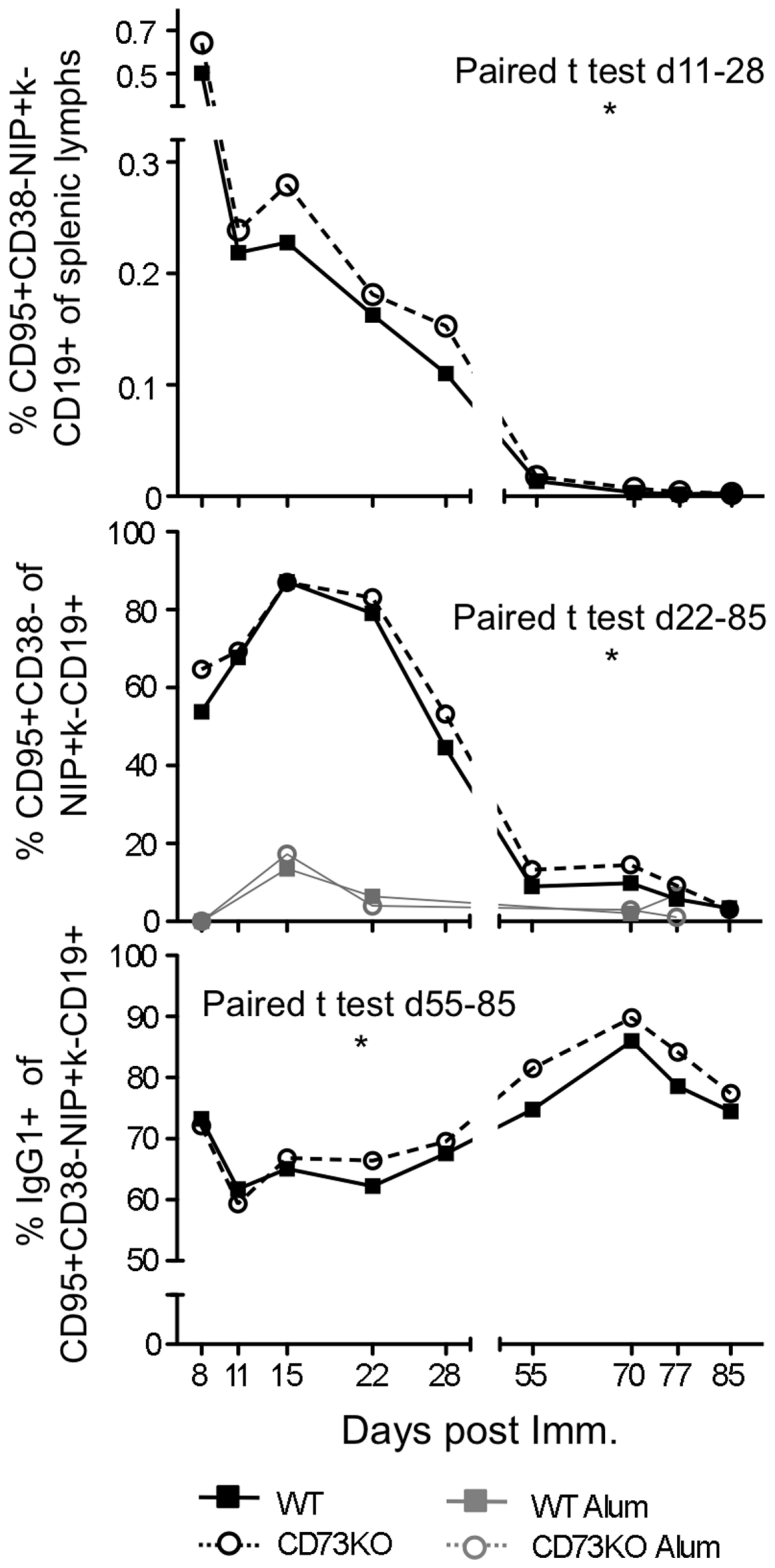
GCs are modestly altered in the absence of CD73. B6 WT (filled squares, solid lines) and CD73KO (empty circles, dashed lines) mice were immunized i.p. with NP-CGG precipitated in alum or alum alone. At the indicated times post immunization, splenocytes were analyzed by flow cytometry. Each point represents pooled data from 3–22 (NP-CGG) or 1–5 (alum alone) individual mice. Day 28 represents 4 independent experiments and day 11 represents 2 independent experiments. 1.3–2 million events were collected per sample. Error bars represent standard deviation of samples. (**Upper panel**) Frequencies of NP-specific GC phenotype B cells (CD95^hi^ CD38^−^ NIP-binding kappa^lo^ CD19^+^) cells among total live lymphocytes. Paired *t* test over d8-28: p = 0.0679. (**Middle panel**) Frequencies of GC phenotype (CD95^hi^ CD38^−^) cells among total NIP-binding kappa^lo^ CD19^+^ B cells. Paired *t* test over d22-85: p = 0.0409. (**Lower panel**) Fraction of IgG_1_ class switched cells among total antigen specific GC B cells. Paired *t* test over days 15–85: p = 0.010.

Given the abundance of CD73 within the GC and the reduction of GC-derived PCs seen in CD73KO mice, we evaluated whether GCs were functionally modulated in the absence of CD73. In CD73KO mice, there was a trend toward expanded antigen-specific GC B cells throughout the time course (paired t-test day 8–85 p = 0.0679) that reached significance during the peak GC, for day 11–28 (paired t-test p = 0.0271; Figure 5, Upper panel). Furthermore, GC B cells comprised a larger fraction of the Ag-specific B cell pool in CD73KO mice in the late GC (day 22 and beyond), (paired t-test for day 22–85 p = 0.0409; Figure 5, Middle panel). We did not observe differences in rates of proliferation or apoptosis, measured by BrdU incorporation and caspase activation [30], [31], [47], [48], to explain these differences (data not shown), though given the small steady state differences, this is not unexpected. Lastly, GCs in CD73KO mice did have a small but consistent and significant increase in the fraction of IgG_1_-switched cells, indicating a qualitative difference in GC function. This was most apparent in the late GC (paired t-test d55-85 p = 0.012; Figure 5, Lower panel).

Since T_FH_ cells express CD73 and since secretion of IL-21 is one of their primary functions, we compared IL-21 mRNA and protein expression among T_FH_ cells from immunized WT and CD73KO mice by qPCR and flow cytometry. There were no detectable differences (Figure S7). Thus, expression of CD73 is not required for production of IL-21 by T_FH_.

### Few alterations in MBC frequency, quality or function in the absence of CD73

**Figure 6:**
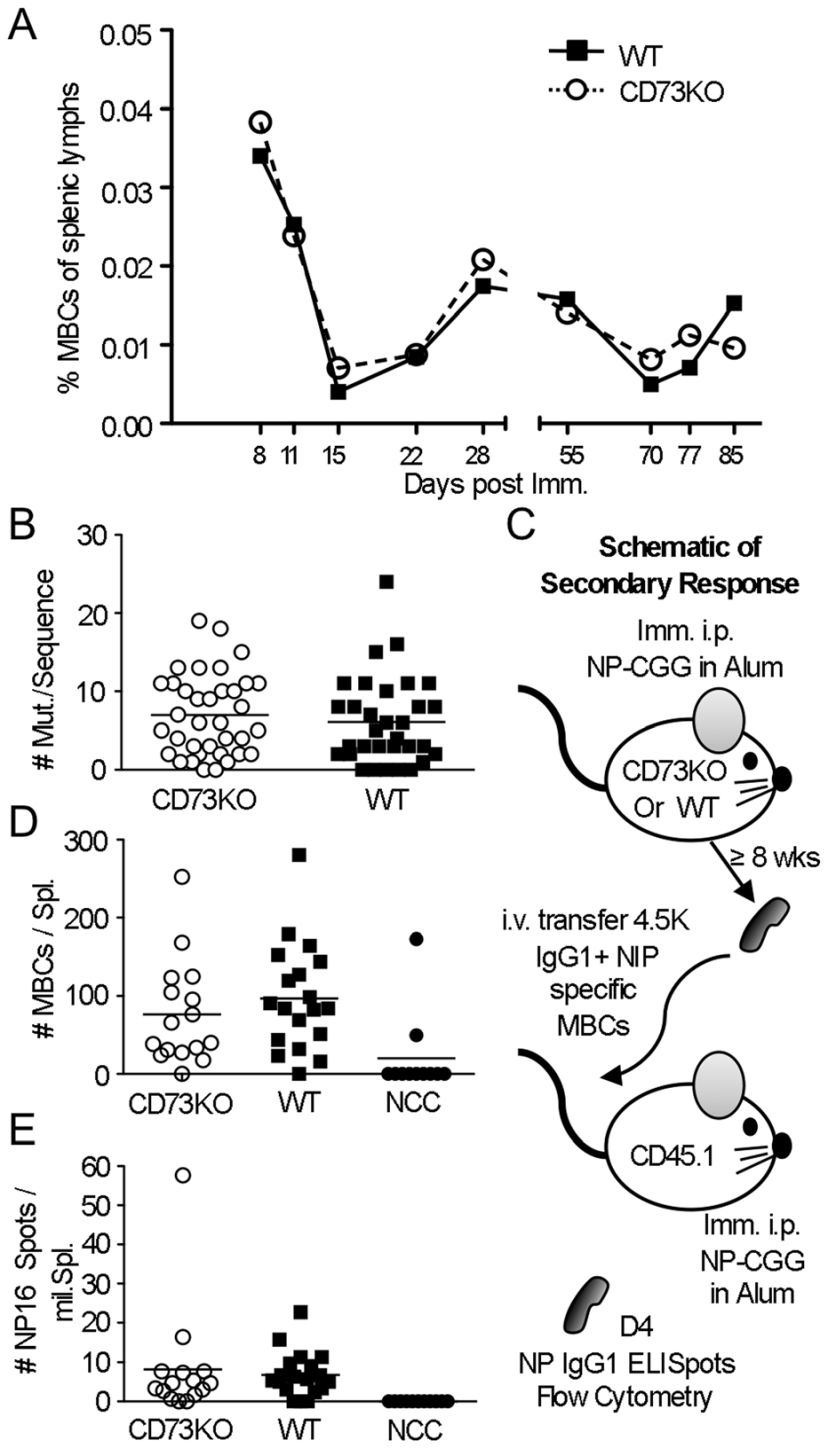
Absence of CD73 does not significantly affect MBCs frequency, quality or function. (**A**) WT (filled squares, solid lines) and CD73KO (empty circles, dashed lines) mice were immunized i.p. with NP-CGG precipitated in alum, and at indicated times post immunization, the splenic MBC compartment was analyzed by FACS. Shown is fraction of live lymphocytes that were IgG_1_-class-switched memory phenotype antigen specific B cells (IgG_1_^+^ CD38^+^ NIP-binding κ^−^ CD19^+^). Day 28 represents 4 independent experiments and day 11 represents 2 independent experiments. 1.3–3 million events were collected per sample. Each point represents pooled data from 3–14 individual mice. Error bars represent standard deviations. (**B**) Comparison of mutational load within Vλ_1_ sequences from IgG_1_^+^ CD38^+^ NIP-binding κ^−^ MBCs purified by FACS from CD73KO (empty circles) and WT (filled squares) spleens. Each point represents an individual Vλ_1_ sequence. Vλ_1_ mutation rates did not significantly differ between groups by Student's *t* test. (**C**) Schematic of experimental design to analyze the secondary response mounted by MBCs formed in the absence of CD73. B6 WT or CD73KO mice were immunized i.p. with NP-CGG in alum. 8 or 12 weeks later, splenocytes were harvested and depleted of T cells. Resulting purified B cells containing 4.5×10^3^ IgG_1_^+^ CD38^+^ NP-specific memory cells were transferred i.v. into CD45.1 congenic recipients. Recipients were i.p. immunized with NP-CGG in alum 16 hours post transfer. Splenocytes were analyzed 4 days later by FACS and ELISpot. (**D**) Numbers of memory phenotype IgG_1_^+^ CD38^+^ NIP-binding splenic B cells after MBC adoptive transfer and secondary immunization. There were no significant differences between groups when analyzed by the Student's *t* test. For comparison, immunized recipient strain non-transferred “no cell control” (NCC) mice are shown (solid circle). (**E**) Frequencies of NP-binding IgG_1_^+^ splenic PCs detected by ELISpot analysis post adoptive transfer and secondary immunization. There were no significant differences between groups (Student's *t* test). Symbols and shading as in D.

CD73 expression defines subsets of MBCs, so we evaluated the effects of loss of CD73 on MBC frequency, quality and function. Isotype-switched MBCs were strictly defined as NP-specific IgG_1_^+^ CD38^+^ CD95^low/int^ B cells, a phenotype that includes antigen-experienced non-GC B cells, not all of which are necessarily destined to become long-lived MBCs [49]. There were no significant differences in MBC frequency over the time course (Figure 6A). Phenotypically, CD73-deficiency did not affect the quality or composition of the MBC compartment, as CD273/PD-L2^+^ and CD80^+^ subsets [20], [25], [48] were present at similar frequencies to WT mice (data not shown). Mutational content in the V-lambda_1_ light chain was also unaffected by CD73-deficiency (Figure 6B). To test whether CD73 affected function of MBC, we performed adoptive transfer and re-immunization. CD73KO MBC gave rise to NP-binding IgG_1_ PB and PCs in numbers and frequencies comparable with WT controls (Figure 6C–E). Thus, in the absence of CD73, MBC are generated at relatively normal frequencies, have an unaltered phenotype, and function normally in the early secondary response.

## Discussion

Our experiments demonstrate that CD73 expression is tightly regulated within the maturing GC, increasing with time, and that CD73 has an important and previously unrecognized role modulating the establishment of the long-lived PC compartment. In contrast, CD73-expressing B and T cells are not abundant in the early GC and neither the early burst of PB/PC nor the early GC are affected measurably by CD73 deficiency.

As CD73 expression marks GC B cells and subsets of MBCs, it was important to ask what role its activity plays in the B cell response and subsequent MBC and PC formation and function. In its absence, we found a relatively specific phenotype of a reduced BM PC compartment. Despite extensive analysis, we additionally detected at most only minor alterations in GCs, and no alterations in early splenic PB/PCs or MBCs. Thus, CD73 activity may not be important for these other arms of the primary response, or other pathways may compensate in its absence.

Together, the data presented here are most consistent with a role for CD73 in supporting the accumulation of BM PCs. Late in the primary response, there was an expected, marked accumulation of BM PCs in WT mice [37] that was blunted in CD73KOs. BM PCs in CD73 KO mice reached a plateau earlier, whereas the numbers continued to increase in WT animals. Were the diminished numbers of BM PCs to reflect a difference in decay rates rather than accumulation, the decay rate would have to be much faster in the KO animals. This in turn would predict that when the accumulation phase was over (i.e. at day 77 and beyond), the CD73KO PCs would continue to decay and then fall-off in numbers. However, this was not observed. These findings suggest that CD73 deficient mice have a defect in PC generation, but once formed, these PCs are stable and long-lived. We cannot completely rule out a deficiency in egress from the spleen or trafficking to or survival in the marrow, but our findings argue against this notion. beta-integrins, CXCR4, CD138, required for their retention in the BM [41], [50], are expressed normally by PCs in the absence of CD73. Further supporting the interpretation that PC survival is independent of CD73 activity, PCs themselves do not express CD73, nor do marrow-resident eosinopils basophils, which support PC survival [42], [43]. While other BM myeloid populations [22], osteoblasts [51], mesenchymal stromal cells [52], [53] and vascular endothelial cells [22] express CD73 and could hypothetically provide required CD73 activity to BM PCs, the fact that diminished PCs were not observed among CD73-deficient recipients of WT BM suggests that any contribution of stromal cell-derived CD73 in the BM to PC survival is minor.

Though CD73 is markedly upegulated on both GC B and T cells, its specific molecular role in the GC remains to be elucidated. This will be a difficult question to address given the complexity of the pathway that CD73 is part of, and since multiple cell types express CD73 and adenosine receptors. However, it is intriguing to consider that CD73 may function in response to hypoxia and increased extracellular ATP generated by the frequent proliferation and apoptosis that define the GC. Consistent with this notion are the observations of hypoxia-inducible factor 1 (HIF-1alpha) expression among human [54] and murine (our unpublished data) GC B cells and the documented role of HIF-1alpha, a master regulator, in promoting CD73 expression [55]. Furthermore, it is clear that levels of extracellular ATP are tightly controlled, and that a biologically prevalent mechanism for reducing ATP in response to cell stress or apoptosis is the conversion to adenosine sequentially by ectonucleoside triphosphate diphosphohydrolase 1 (Entpd1, CD39) and related ectonucleosides and then generation of adenosine from these intermediates via CD73 [1]–[3]. The modulated expression—observed in our gene expression microarray studies—of CD39 and ectonucleosidases Entpd4, Entdp5, Entdp7 the adenosine receptor A2a and CD73 seen among GC and MBCs ([27] and unpublished data) intriguingly suggests the hypothesis that this pathway is active in the GC microenvironment.

Taken together, our data suggest an important and previously underappreciated role for ATP metabolism and adenosine signaling in regulating the B cell response to antigen, and specifically a role for the ecto-5′nucleotidase CD73 in modulating late GC reaction and the development of long-lived PCs. These mechanisms are likely to be of broader immunologic significance given the striking parallels in expression patterns between mouse and human B and T cell subpopulations. These findings provide new insights into the regulation of long-term humoral immunity development and suggest pathways by which pathogen-induced, inflammation and hypoxia, as well as genetic variation, may shape the B cell response via CD73-dependent immunomodulation.

## Supporting Information

Figure S1 **FACS gating strategy for B cell subpopulations.** RBC-depleted splenocytes or BM cells were stained and analyzed by flow cytometry. Representative FACS histograms and dot plots demonstrating gating strategies are shown. Live, single cells were first gated by a combination of forward and side scatter profiles and by EMA exclusion. (**A**) GC B cell and MBC gating. Shown are splenocytes 28 days post immunization with NP-CGG in alum i.p. Cells were stained with reagents to identify expression of CD19, CD38, CD95, IgG_1_ and Igkappa and NIP-binding, as detailed in Material and Methods. (**B**) PB/PC gating. Shown are BM cells 28 days and splenocytes 11 days post immunization with NP-CGG in alum i.p. Cells were stained with reagents to identify expression of B220, CD138/Syndecan, and for intracellular NIP-binding, as detailed in Material and Methods. (**C**) TFH gating. Shown are splenocytes 11 days post immunization with NP-CGG in alum i.p. Cells were stained with reagents to identify expression of TCRbeta, CD4, CD44, PD1, and ICOS as detailed in Material and Methods.
(TIF)

Figure S2 **CD73 is not expressed by BM eosinophils or basophils.** BM cells from WT mice immunized with NP-CGG in alum i.p. 28-days previously were stained and analyzed by flow cytometry. Representative FACS histograms are shown. Live, single cells were first gated by EMA exclusion. (**A**) Basophil profiles. Basophils were identified by high surface expression of Siglec-F and F4/80 and intermediate expression of CD11b. Shown are CD73 (heavy line) and isotype control (heavy shading) stained basophils. (**B**) Eosinophil profiles. Eosinophils were identified by high surface expression of CD49b and IgE. Shown are CD73 (heavy line) and isotype control (heavy shading) stained eosinophils.
(TIF)

Figure S3 **Splenic myeloid compartments are relatively unaffected by the absence of CD73.** At the indicated days pre or post i.p. immunization with NP-CGG in alum, spleens from B6 WT and CD73KO control mice were stained and analyzed by flow cytometry. (**A**) CD73 expression on the indicated cell types from unimmunized spleens of WT (solid line) and CD73KO (shaded gray) mice. (**B**) Absolute numbers cDCs, pDCs, neutrophils and macrophages per spleen. Macrophages were identified as Gr1^int/low^ F4/80^+^ CD11b^+^ CD19^−^, cDCs as CD11c^+^ IA/IE^+^ CD19^−^, pDCs as SiglecH^+^ CD317(BST2)^+^ CD19^−^ and neutrophils as CD11b^+^ Ly6g^+^ CD19^−^ live cells. Each point represents the average of 5–10 individual spleens. Error bars depict standard deviations. * and ** indicate Student's *t*-test p values of <0.05 and <0.01, respectively. WT is shown as filled squares with solid lines and CD73KO as empty circles with dashed lines.
(TIF)

Figure S4 **Evaluation of extent chimerism of mice depicted in** **Figure 4** **.** Chimeric animals were established from adoptive transfer of WT or CD73KO donor BM into irradiated WT or CD73KO hosts. Donor and hosts were allotypically distinct (CD45.1 and CD45.2) in all chimeric combinations except the CD73KO donor/CD73KO host controls. 6-weeks post BM transfer, chimeric animals were immunized i.p. with NP-CGG in alum and euthanized 11-weeks later. Quality of chimerism was evaluated by flow cytometric analysis of CD45.2 frequency among splenic B and T cells; 1–3 million events were collected per sample. Data shown are pooled from 6 (WT into WT), 5 (KO into WT) and 1 (WT into KO and KO into KO) individual experiments, each with 4–10 mice per group. Each point represents an individual mouse. Mean values are depicted by heavy horizontal lines. (**Top panel**) Percent of splenic B cells expressing CD45.2. (**Bottom panel**) Percent of splenic T cells expressing CD45.2.
(TIF)

Figure S5 **Neither B nor T cell derived CD73 alone is required for establishment of the BM PC compartment.** (**A**) Schematic of experimental design. Chimeric animals were established from adoptive transfer of the depicted combinations and ratios of WT, CD73KO, muMT and TCRbetaKO donor BM into irradiated WT hosts. Donor and hosts were allotypically distinct (CD45.2 and CD45.1, respectively). 6-weeks post BM transfer, chimeric animals were immunized i.p. with NP-CGG in alum, and 11-weeks later, BM PCs were enumerated by ELISpot analysis. Extent of chimerism was evaluated by flow cytometric analysis of CD45.2 expression by splenic B and T cells. (**B**) Evaluation of chimeric mice 11-weeks post-immunization. Each point represents an individual mouse. Data shown are pooled from 3 individual experiments. Mean values are depicted by heavy horizontal lines. (**Top panel**) Percent of splenic B cells expressing the donor CD45.2 allele. (**Middle panel**) Percent of splenic T cells expressing the donor CD45.2 allele. (**Bottom panel**) Frequency of IgG_1_ NP-specific PCs per million BM cells, determined by ELISpot analysis.
(TIF)

Figure S6 **In the absence of CD73, splenic expansion post immunization is reduced, but nucleated cell number and composition are unaffected.** B6 WT (filled squares, solid line) and CD73KO (empty circles, dashed line) mice were immunized i.p. with NP-CGG precipitated in alum. At the indicated times post immunization, spleens were harvested and analyzed. Each point represents the average of 3–10 individual spleens. Error bars depict standard deviations. (**A**) Splenic weights. (**B**) Absolute numbers of nucleated cells per spleen after RBC lysis. (**C**) Absolute numbers of B (top), T (middle) and CD4+ T (bottom) lymphocytes per spleen, as determined by flow cytometric analysis of CD19^+^, CD3epsilon^+^ and CD3epsilon^+^CD4^+^ live cells, respectively.
(TIF)

Figure S7 **IL-21 expression by T_FH_ cells is unaltered in the absence of CD73.** 28- and 29-days post i.p immunization with NP-CGG in alum, splenic T_FH_ cells from CD73KO (open circles) and WT mice (filled squares) were analyzed for IL-21 mRNA or protein expression. mRNA and protein experiments were conducted separately, and each point represents an individual mouse. (**A**) Live PI-excluding TCRbeta^+^ CD4^+^ CD44^+^ CCR7^−^ ICOS^+^ CXCR5^+^ T_FH_ cells were sorted on a BD FACSAria. Total RNA was isolated with the Allprep DNA/RNA mini kit (Qiagen, Valencia, CA) and cDNA synthesized and qPCR with SYBR Green performed as previously described (44). IL-21 primer sequences were: sense, 5′-TGAAAGCCTGTGGAAGTGC AAACC-3′, and antisense, 5′-AGCAGATTCATCACAGGACACCCA-3′ (39). IL-21 and beta-actin products were amplified from identical cDNA cell equivalents. Shown is relative amplification of IL-21 cDNA normalized to beta-Actin expression, expressed as beta-Actin threshold cycle (C_t_) minus IL-21 C_t_ (Student's t-test p = 0.9236). Shown is one of two similar experimental replicates with 4–5 individual mice per group. (**B**) For flow cytometric analysis of IL-21 protein expression, splenocytes were stimulated in vitro for 5 hours with phorbol-12-myristate-13-acetate (PMA; 20 ng/ml; EMD Millipore, Billerica, MA) and ionomycin (750 ng/mL; EMD Millipore, Billerica, MA). After 1-hour, transport out the endoplasmic reticulum was inhibited by the addition of Brefeldin A (Biolegend, San Diego, CA), per the manufacture's instructions. Post stimulation, splenocytes were stained for surface markers, permeabilized with Perm/Wash Buffer (BD Biosciences), incubated with 10% goat and rat serum followed with recombinant Mouse IL-21R Fc Chimera (R&D Systems, Minneapolis, MN) and finally PE goat-F(ab′)2 -anti-human IgG-Fc (Jackson ImmunoResearch, West Grove, PA). T_FH_ cells were gated as EMA^−^TCRbeta^+^ CD4^+^ CD44^+^ PD1^+^ ICOS^+^. Shown are the percent of T_FH_ cells that express IL-21 protein among stimulated, unstimulated and secondary staining-only control mice (10, 2 and 10 replicates per group, respectively). Student's t-test of CD73KO and WT stimulated samples yielded p-value of 0.7971. (**C**) Median fluorescence intensity (MFI) of IL-21 expression among IL21^+^ T_FH_ cells, identified in (B). Student's t-test p-value of 0.4150.
(TIF)
