# Supplementary figures and images for: CD73 Expression Is Dynamically Regulated in the Germinal Center and Bone Marrow Plasma Cells Are Diminished in Its Absence
Source: PLoS One. 2014 Mar 24;9(3):e92009. doi: 10.1371/journal.pone.0092009 (PMC3963874; doi:10.1371/journal.pone.0092009)

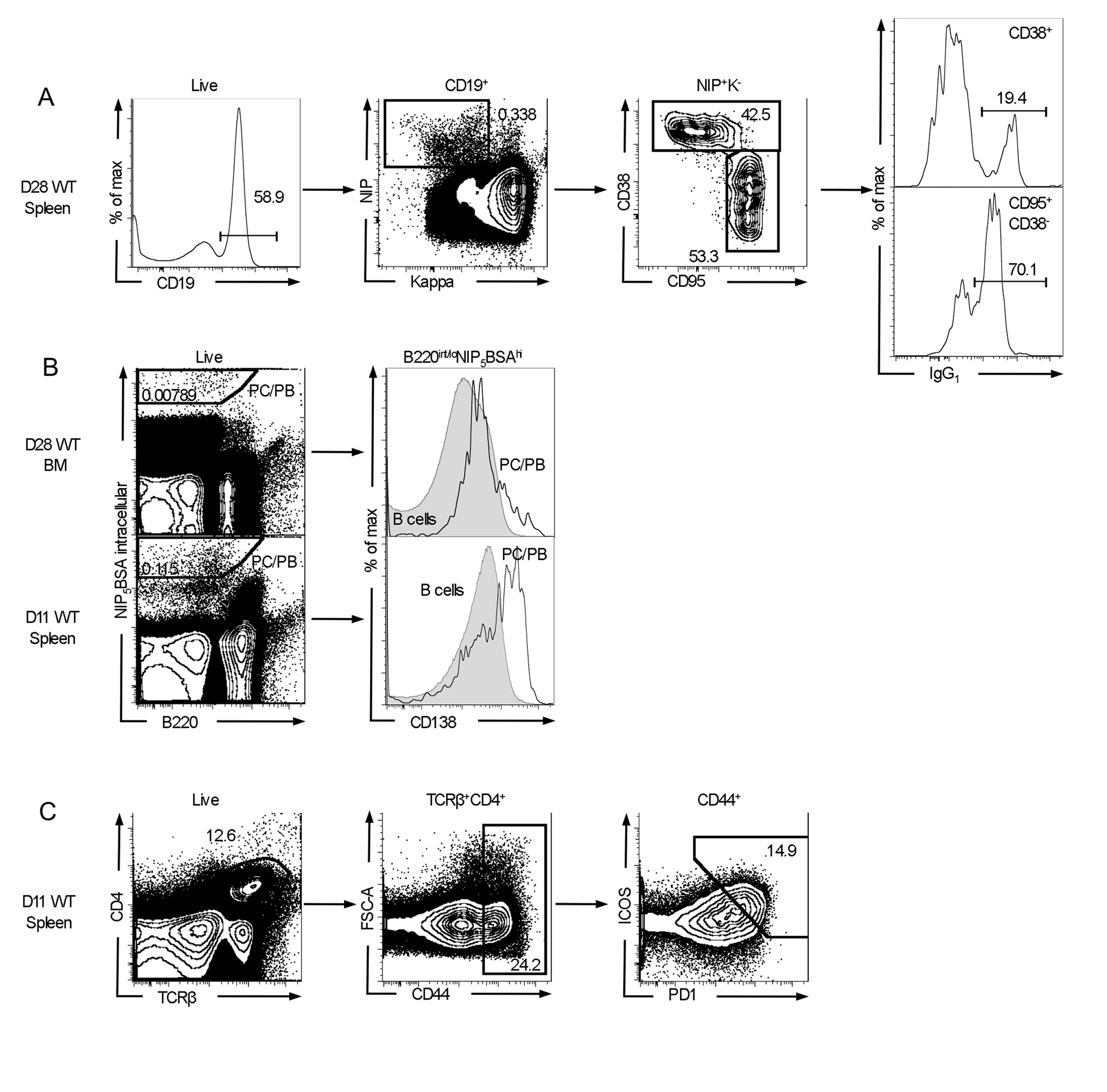

Supplement: Figure S1 — FACS gating strategy for B cell subpopulations. RBC-depleted splenocytes or BM cells were stained and analyzed by flow cytometry. Representative FACS histograms and dot plots demonstrating gating strategies are shown. Live, single cells were first gated by a combination of forward and side scatter profiles and by EMA exclusion. (A) GC B cell and MBC gating. Shown are splenocytes 28 days post immunization with NP-CGG in alum i.p. Cells were stained with reagents to identify expression of CD19, CD38, CD95, IgG1 and Igkappa and NIP-binding, as detailed in Material and Methods. (B) PB/PC gating. Shown are BM cells 28 days and splenocytes 11 days post immunization with NP-CGG in alum i.p. Cells were stained with reagents to identify expression of B220, CD138/Syndecan, and for intracellular NIP-binding, as detailed in Material and Methods. (C) TFH gating. Shown are splenocytes 11 days post immunization with NP-CGG in alum i.p. Cells were stained with reagents to identify expression of TCRbeta, CD4, CD44, PD1, and ICOS as detailed in Material and Methods. (TIF) [file pone.0092009.s001.tif]

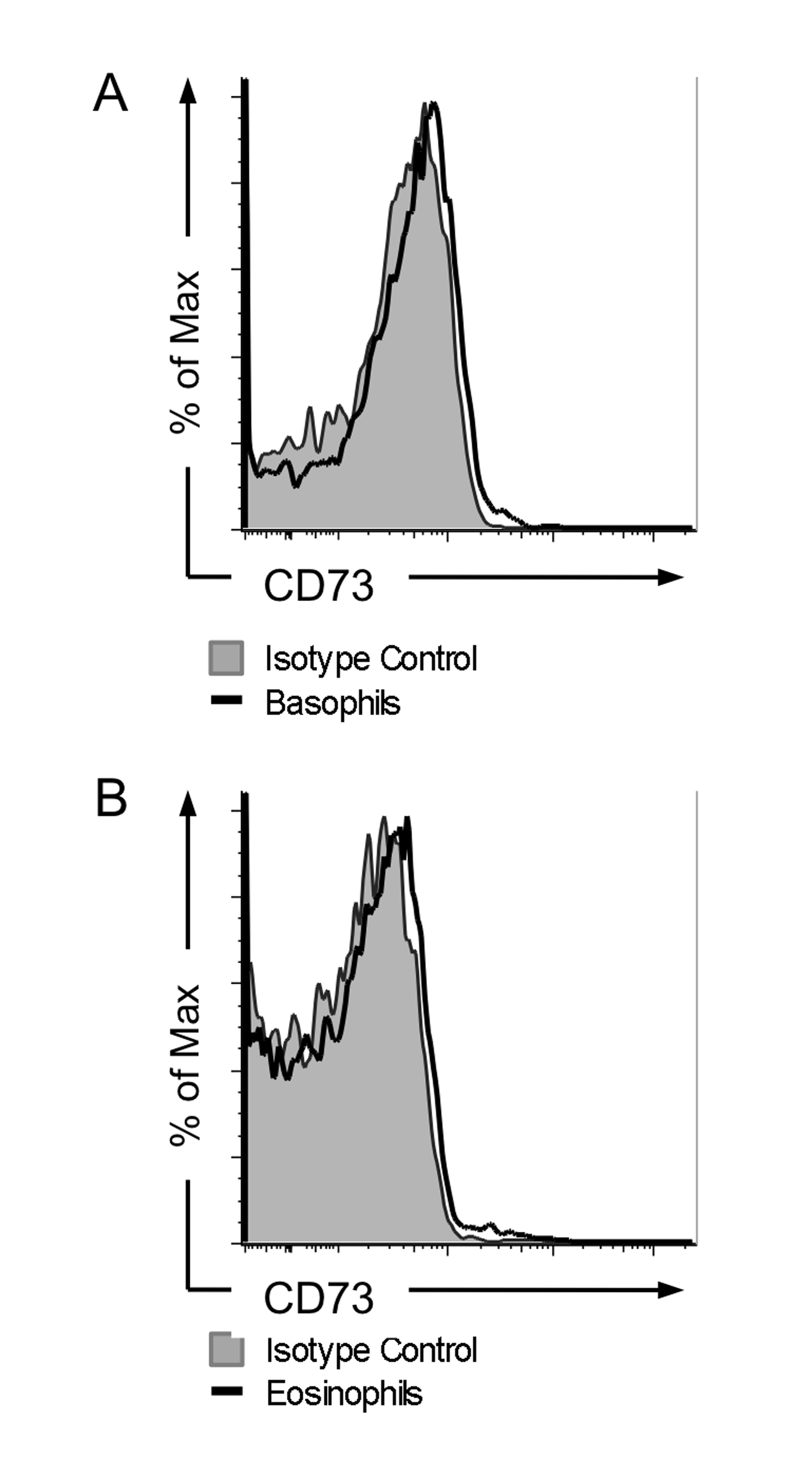

Supplement: Figure S2 — CD73 is not expressed by BM eosinophils or basophils. BM cells from WT mice immunized with NP-CGG in alum i.p. 28-days previously were stained and analyzed by flow cytometry. Representative FACS histograms are shown. Live, single cells were first gated by EMA exclusion. (A) Basophil profiles. Basophils were identified by high surface expression of Siglec-F and F4/80 and intermediate expression of CD11b. Shown are CD73 (heavy line) and isotype control (heavy shading) stained basophils. (B) Eosinophil profiles. Eosinophils were identified by high surface expression of CD49b and IgE. Shown are CD73 (heavy line) and isotype control (heavy shading) stained eosinophils. (TIF) [file pone.0092009.s002.tif]

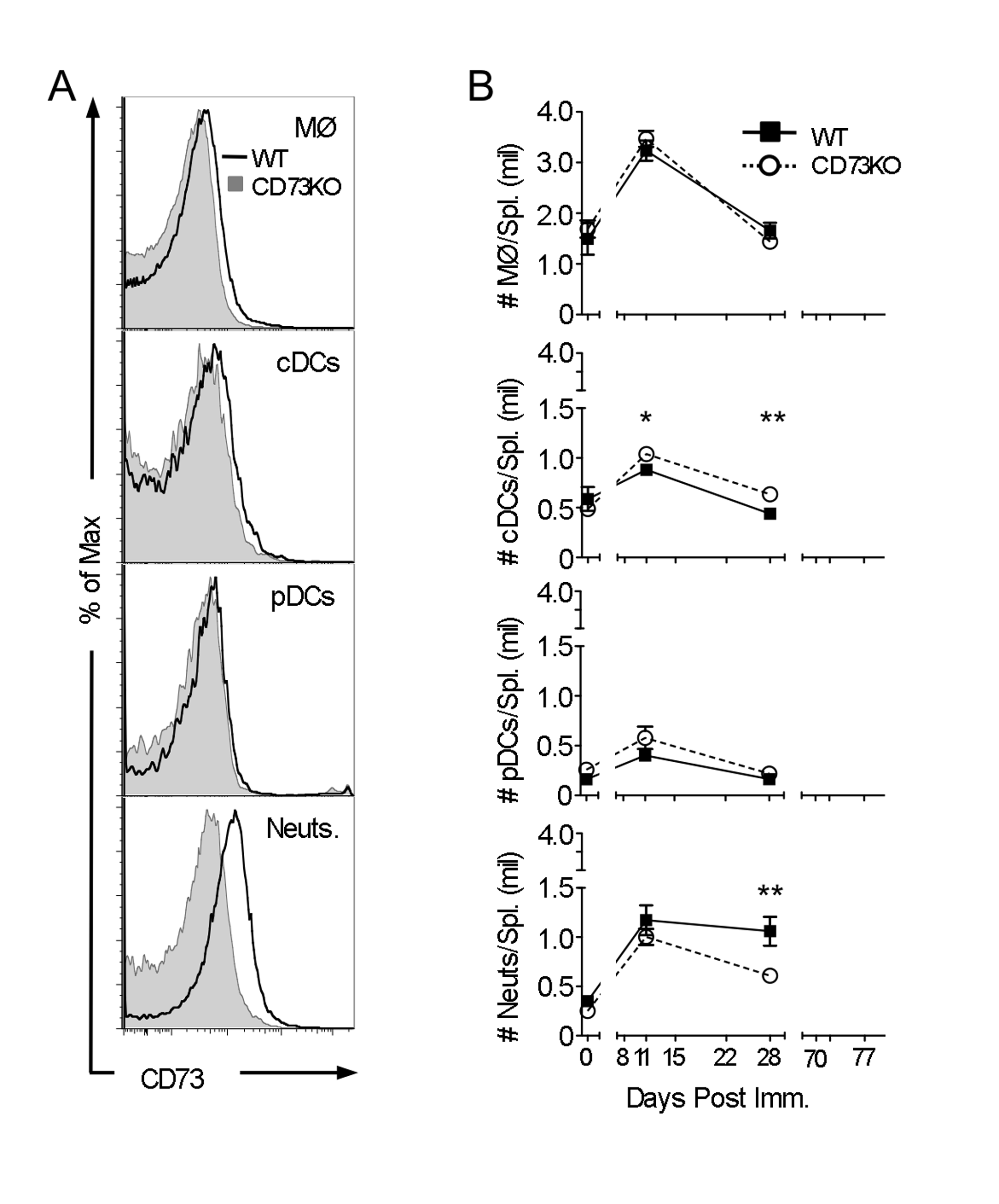

Supplement: Figure S3 — Splenic myeloid compartments are relatively unaffected by the absence of CD73. At the indicated days pre or post i.p. immunization with NP-CGG in alum, spleens from B6 WT and CD73KO control mice were stained and analyzed by flow cytometry. (A) CD73 expression on the indicated cell types from unimmunized spleens of WT (solid line) and CD73KO (shaded gray) mice. (B) Absolute numbers cDCs, pDCs, neutrophils and macrophages per spleen. Macrophages were identified as Gr1int/low F4/80+ CD11b+ CD19−, cDCs as CD11c+ IA/IE+ CD19−, pDCs as SiglecH+ CD317(BST2)+ CD19− and neutrophils as CD11b+ Ly6g+ CD19− live cells. Each point represents the average of 5–10 individual spleens. Error bars depict standard deviations. * and ** indicate Student's t-test p values of <0.05 and <0.01, respectively. WT is shown as filled squares with solid lines and CD73KO as empty circles with dashed lines. (TIF) [file pone.0092009.s003.tif]

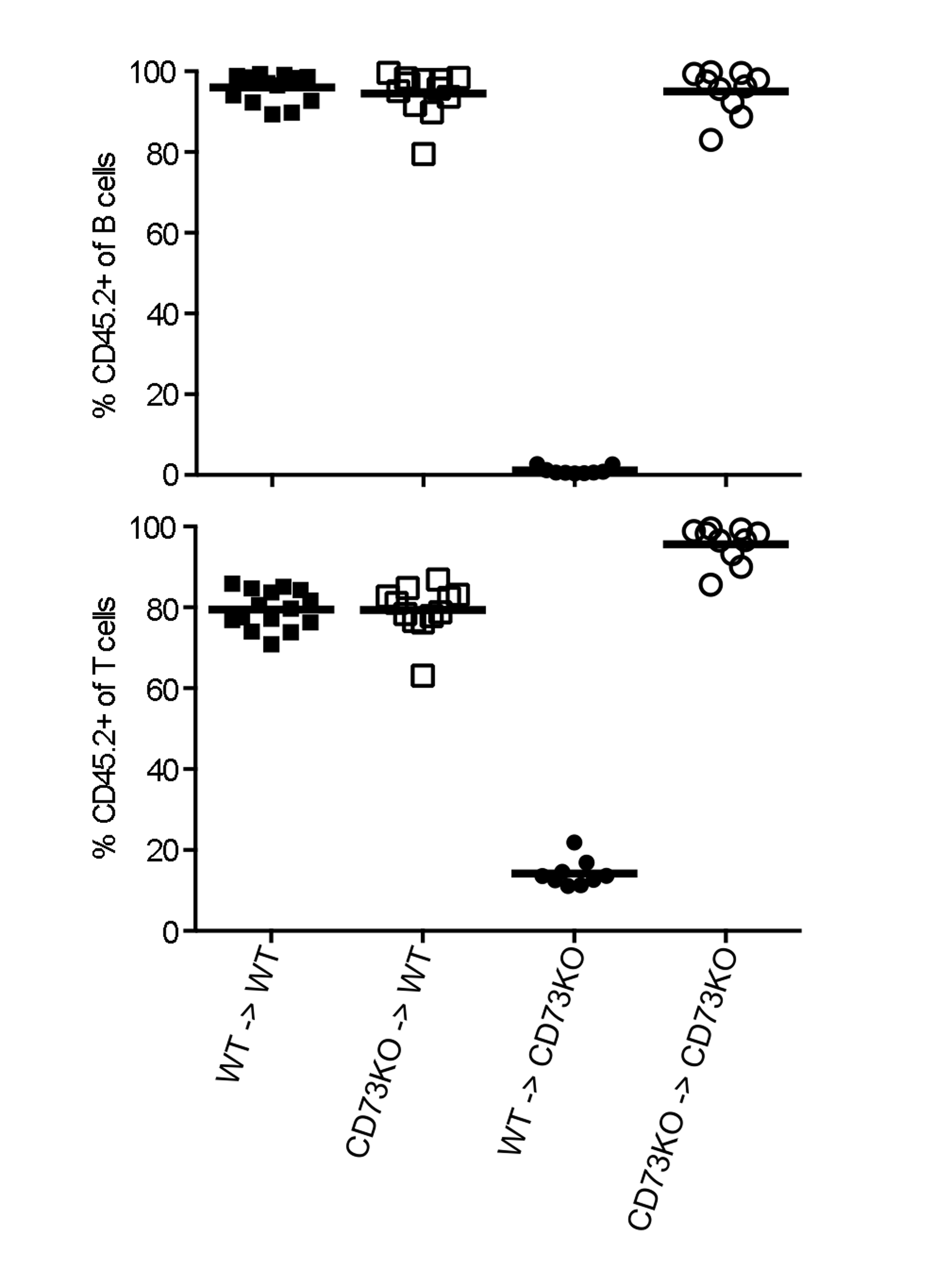

Supplement: Figure S4 — Evaluation of extent chimerism of mice depicted in Figure 4 . Chimeric animals were established from adoptive transfer of WT or CD73KO donor BM into irradiated WT or CD73KO hosts. Donor and hosts were allotypically distinct (CD45.1 and CD45.2) in all chimeric combinations except the CD73KO donor/CD73KO host controls. 6-weeks post BM transfer, chimeric animals were immunized i.p. with NP-CGG in alum and euthanized 11-weeks later. Quality of chimerism was evaluated by flow cytometric analysis of CD45.2 frequency among splenic B and T cells; 1–3 million events were collected per sample. Data shown are pooled from 6 (WT into WT), 5 (KO into WT) and 1 (WT into KO and KO into KO) individual experiments, each with 4–10 mice per group. Each point represents an individual mouse. Mean values are depicted by heavy horizontal lines. (Top panel) Percent of splenic B cells expressing CD45.2. (Bottom panel) Percent of splenic T cells expressing CD45.2. (TIF) [file pone.0092009.s004.tif]

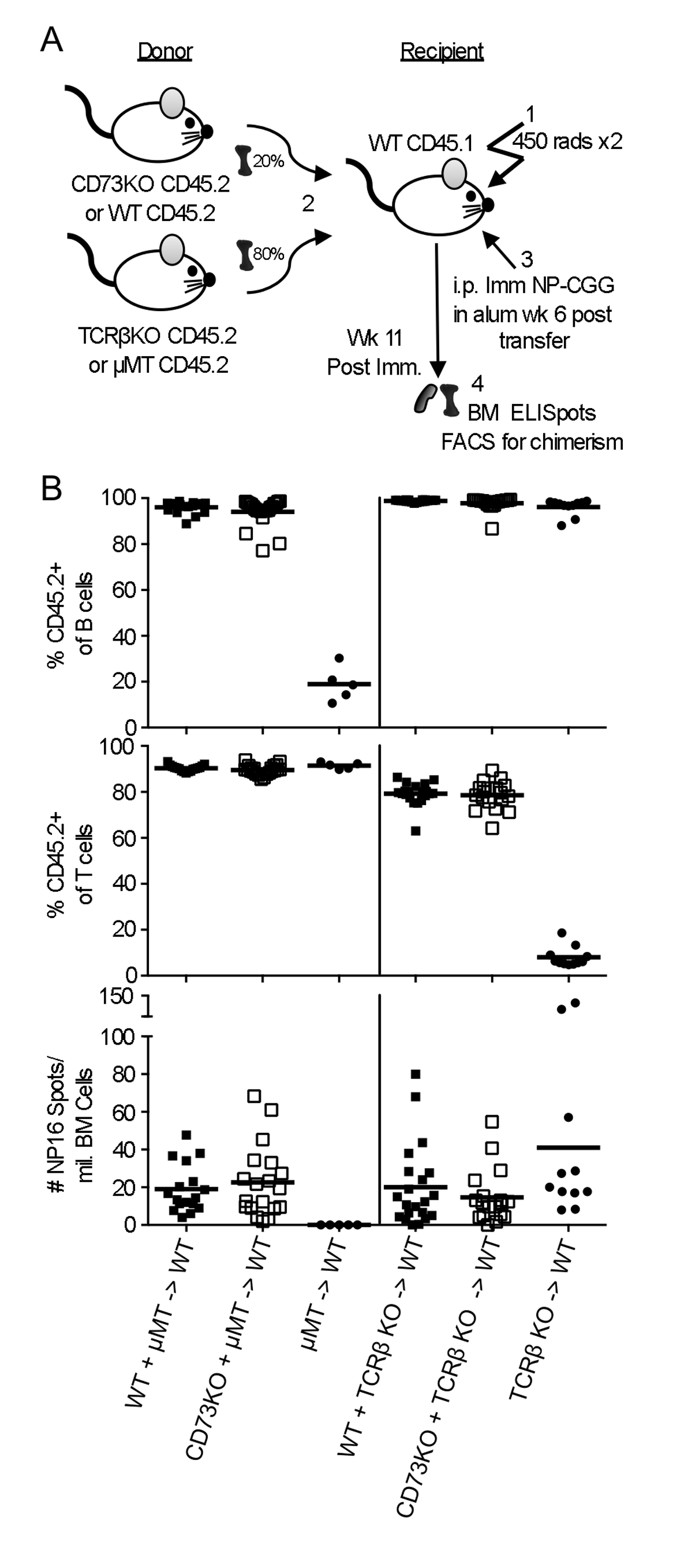

Supplement: Figure S5 — Neither B nor T cell derived CD73 alone is required for establishment of the BM PC compartment. (A) Schematic of experimental design. Chimeric animals were established from adoptive transfer of the depicted combinations and ratios of WT, CD73KO, muMT and TCRbetaKO donor BM into irradiated WT hosts. Donor and hosts were allotypically distinct (CD45.2 and CD45.1, respectively). 6-weeks post BM transfer, chimeric animals were immunized i.p. with NP-CGG in alum, and 11-weeks later, BM PCs were enumerated by ELISpot analysis. Extent of chimerism was evaluated by flow cytometric analysis of CD45.2 expression by splenic B and T cells. (B) Evaluation of chimeric mice 11-weeks post-immunization. Each point represents an individual mouse. Data shown are pooled from 3 individual experiments. Mean values are depicted by heavy horizontal lines. (Top panel) Percent of splenic B cells expressing the donor CD45.2 allele. (Middle panel) Percent of splenic T cells expressing the donor CD45.2 allele. (Bottom panel) Frequency of IgG1 NP-specific PCs per million BM cells, determined by ELISpot analysis. (TIF) [file pone.0092009.s005.tif]

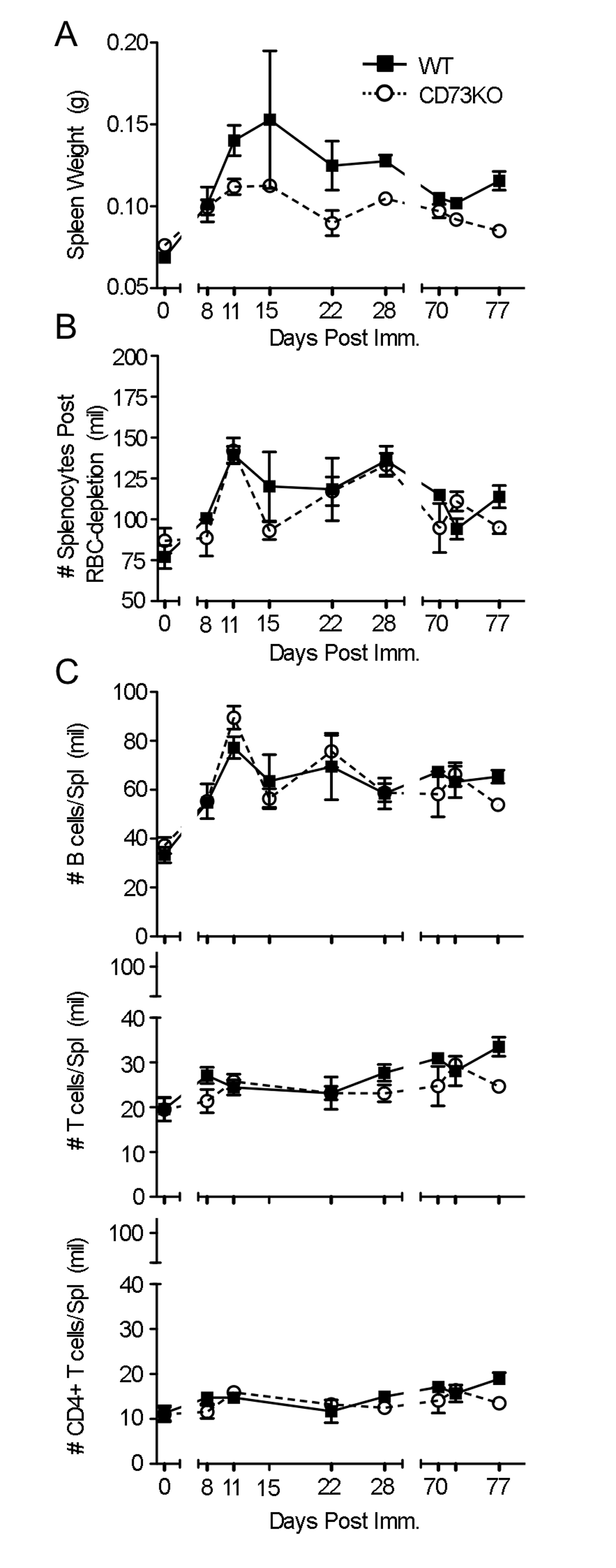

Supplement: Figure S6 — In the absence of CD73, splenic expansion post immunization is reduced, but nucleated cell number and composition are unaffected. B6 WT (filled squares, solid line) and CD73KO (empty circles, dashed line) mice were immunized i.p. with NP-CGG precipitated in alum. At the indicated times post immunization, spleens were harvested and analyzed. Each point represents the average of 3–10 individual spleens. Error bars depict standard deviations. (A) Splenic weights. (B) Absolute numbers of nucleated cells per spleen after RBC lysis. (C) Absolute numbers of B (top), T (middle) and CD4+ T (bottom) lymphocytes per spleen, as determined by flow cytometric analysis of CD19+, CD3epsilon+ and CD3epsilon+CD4+ live cells, respectively. (TIF) [file pone.0092009.s006.tif]

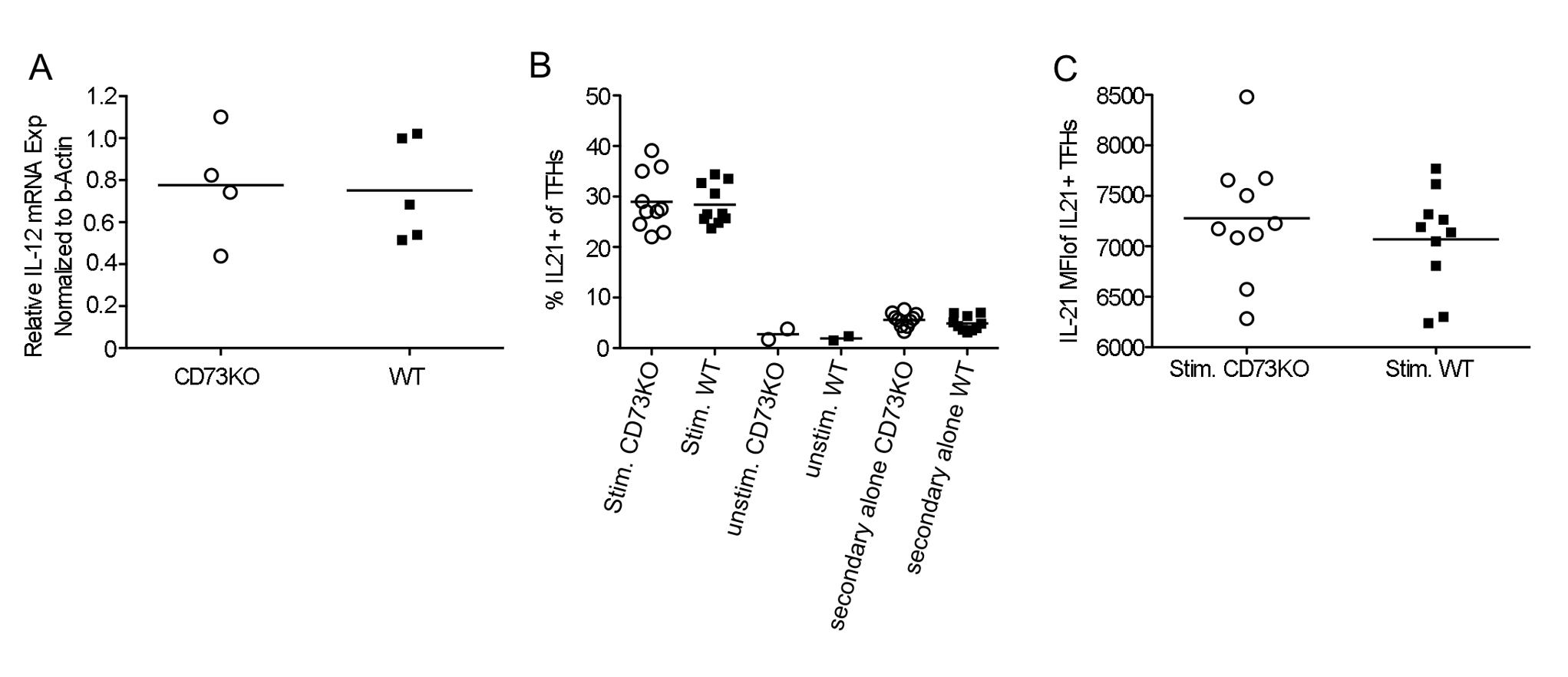

Supplement: Figure S7 — IL-21 expression by TFH cells is unaltered in the absence of CD73. 28- and 29-days post i.p immunization with NP-CGG in alum, splenic TFH cells from CD73KO (open circles) and WT mice (filled squares) were analyzed for IL-21 mRNA or protein expression. mRNA and protein experiments were conducted separately, and each point represents an individual mouse. (A) Live PI-excluding TCRbeta+ CD4+ CD44+ CCR7− ICOS+ CXCR5+ TFH cells were sorted on a BD FACSAria. Total RNA was isolated with the Allprep DNA/RNA mini kit (Qiagen, Valencia, CA) and cDNA synthesized and qPCR with SYBR Green performed as previously described (44). IL-21 primer sequences were: sense, 5′-TGAAAGCCTGTGGAAGTGC AAACC-3′, and antisense, 5′-AGCAGATTCATCACAGGACACCCA-3′ (39). IL-21 and beta-actin products were amplified from identical cDNA cell equivalents. Shown is relative amplification of IL-21 cDNA normalized to beta-Actin expression, expressed as beta-Actin threshold cycle (Ct) minus IL-21 Ct (Student's t-test p = 0.9236). Shown is one of two similar experimental replicates with 4–5 individual mice per group. (B) For flow cytometric analysis of IL-21 protein expression, splenocytes were stimulated in vitro for 5 hours with phorbol-12-myristate-13-acetate (PMA; 20 ng/ml; EMD Millipore, Billerica, MA) and ionomycin (750 ng/mL; EMD Millipore, Billerica, MA). After 1-hour, transport out the endoplasmic reticulum was inhibited by the addition of Brefeldin A (Biolegend, San Diego, CA), per the manufacture's instructions. Post stimulation, splenocytes were stained for surface markers, permeabilized with Perm/Wash Buffer (BD Biosciences), incubated with 10% goat and rat serum followed with recombinant Mouse IL-21R Fc Chimera (R&D Systems, Minneapolis, MN) and finally PE goat-F(ab′)2 -anti-human IgG-Fc (Jackson ImmunoResearch, West Grove, PA). TFH cells were gated as EMA−TCRbeta+ CD4+ CD44+ PD1+ ICOS+. Shown are the percent of TFH cells that express IL-21 protein among stimulated, unstimulated and secondary stain [file pone.0092009.s007.tif]
